# NextPBM: a platform to study cell-specific transcription factor binding and cooperativity

**DOI:** 10.1093/nar/gkz020

**Published:** 2019-01-18

**Authors:** Nima Mohaghegh, David Bray, Jessica Keenan, Ashley Penvose, Kellen K Andrilenas, Vijendra Ramlall, Trevor Siggers

**Affiliations:** 1 Department of Biology and Biological Design Center, Boston University, Boston, MA, USA; 2 Bioinformatics Program, Boston University, Boston, MA, USA

## Abstract

High-throughput (HT) *in vitro* methods for measuring protein-DNA binding have become invaluable for characterizing transcription factor (TF) complexes and modeling gene regulation. However, current methods do not utilize endogenous proteins and, therefore, do not quantify the impact of cell-specific post-translational modifications (PTMs) and cooperative cofactors. We introduce the HT nextPBM (nuclear extract protein-binding microarray) approach to study DNA binding of native cellular TFs that accounts for PTMs and cell-specific cofactors. We integrate immune-depletion and phosphatase treatment steps into our nextPBM pipeline to characterize the impact of cofactors and phosphorylation on TF binding. We analyze binding of PU.1/SPI1 and IRF8 from human monocytes, delineate DNA-sequence determinants for their cooperativity, and show how PU.1 affinity correlates with enhancer status and the presence of cooperative and collaborative cofactors. We describe how nextPBMs, and our accompanying computational framework, can be used to discover cell-specific cofactors, screen for synthetic cooperative DNA elements, and characterize TF cooperativity.

## INTRODUCTION

Defining the principles that govern transcription factor (TF) binding and the assembly of multi-protein TF complexes remains a challenge (1,2). High-throughput (HT) *in vitro* techniques (both microarray- and sequencing-based) exist to characterize the DNA binding of TFs (2,3) and cooperative TF complexes (4–6). Current approaches assay the binding of purified or *in vitro* produced protein samples (5,7,8), or tagged protein overexpressed in cells (e.g. HEK293) (9,10). Consequently, these approaches do not assay the impact of cell-specific post-translational modifications (PTMs), which are known to have diverse effects on TF binding and function (11,12), and do not account for the impact of cell-specific cofactors that can bind cooperatively with TFs.

**Figure 1.**
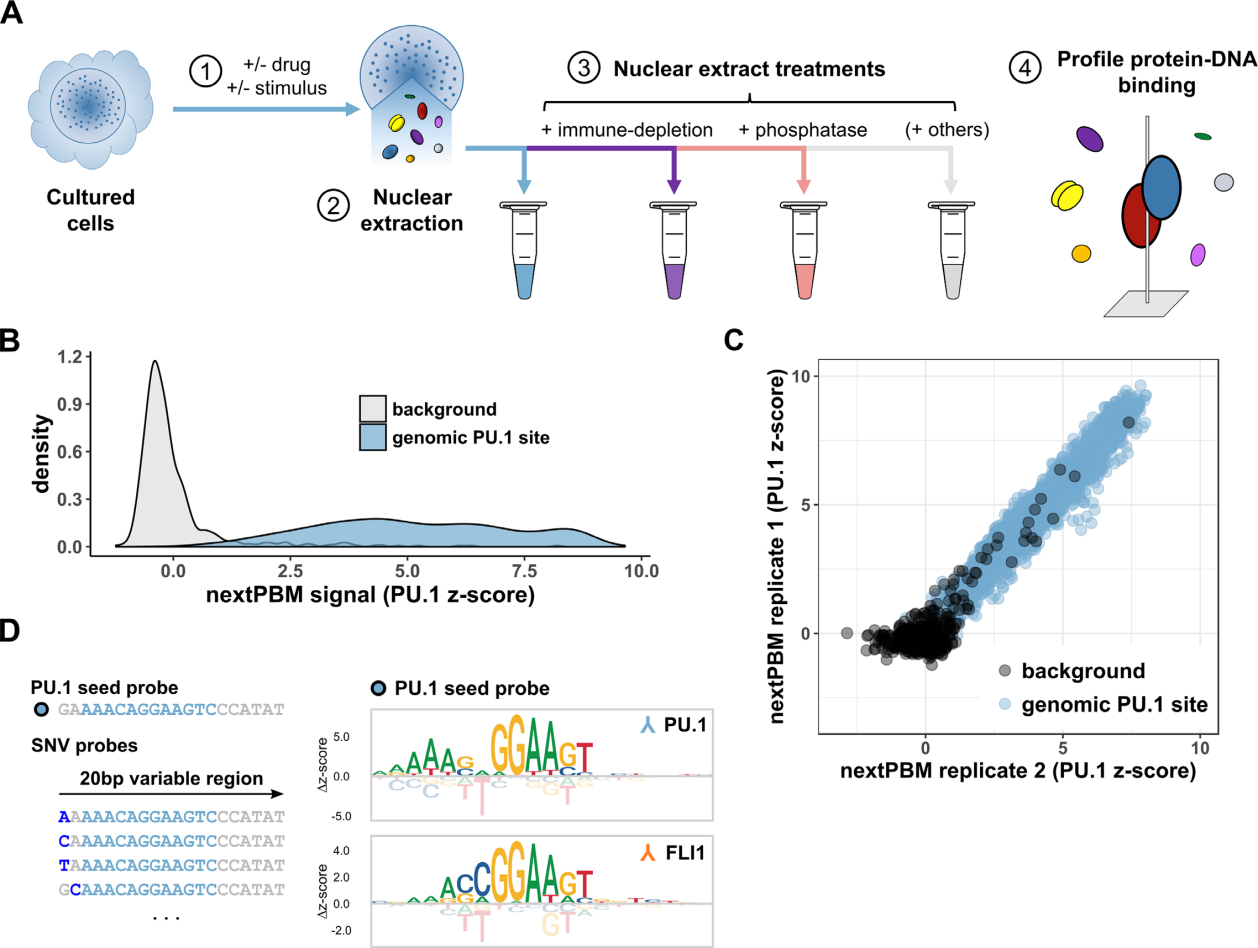
Nuclear extract protein-binding microarrays (nextPBMs). (**A**) Workflow schematic for the nextPBM protocol. (1) Cultured cells can be stimulated or treated with a drug prior to nuclear extraction. (2) Total soluble protein content is harvested from cell nuclei using an optimized protocol (see Materials and Methods). (3) Nuclear extract can be treated in parallel enzymatically (i.e. by phosphatase treatment) and components of interest can be depleted (i.e. by immune-depletion using a targeted antibody) depending on goals of the experiments. 4) DNA binding affinity of one or more transcription factors of interest are profiled in parallel directly from nuclear extract. (**B**) Density of PU.1 nextPBM *z*-scores obtained at random background probes (*n* = 500) and at genomic PU.1 binding sites (*n* = 2615). (**C**) Scatterplot of PU.1 binding *z*-scores obtained by DNA probes corresponding to random background (black) and genomic PU.1 sites (blue) in different biological replicates. (**D**) Left: Schematic representation of the single nucleotide variant (SNV) probes corresponding to an example PU.1 seed probe. Genomic sequence corresponding to the PU.1 motif is highlighted in sky blue within a larger 20bp sequence. SNVs within a given SNV probe are shown in dark blue. Right: Sequence logos obtained for the same genomic PU.1 seed probe using a PU.1 antibody (top) and an FLI1 antibody (bottom). Δz-scores are computed relative to the median score obtained within a given column.

To characterize cell-specific TF binding features and account for the impact of cofactors and PTMs, we have developed nextPBMs (nuclear extract protein-binding microarrays) (Figure 1A). PBMs are double-stranded DNA microarrays that allow *in vitro* measurement of protein binding to tens of thousands of unique DNA sequences (7). NextPBM extends the PBM methodology by using total nuclear extracts in place of purified, IVT, or over-expressed proteins (Materials and Methods). To test the impact of specific cofactors and PTMs on binding, we have developed immune-depletion and phosphatase treatment steps into our nextPBM pipeline (Figure 1A). We describe a computational framework based on binding to single-nucleotide variant (SNV) sites that provides a powerful approach to study DNA-binding specificity and protein cooperativity when assaying heterogenous NEs. We use nextPBMs to analyze the DNA binding of the myeloid cell-lineage factors PU.1 and IRF8, and discuss our results. We outline how nextPBMs can be used to discover cooperative TF binding and to infer the identity of cooperative-acting factors. Finally, we demonstrate how nextPBMs can be used to screen for cooperatively bound synthetic DNA elements. NextPBMs are an extendible and robust HT method to assay the binding of proteins to genomic or synthetic sites that can capture the impact of cell-specific cofactors and PTMs on TF-DNA binding.

## MATERIALS AND METHODS

### Cell culture

THP-1 cells were purchased from ATCC (cat # TIB-202) and cultured in RPMI 1640 media with 10% FBS supplemented by 50 unit/ml Penicillin and 50 μg/ml Streptomycin. HEK-293T cells for Lentivirus packaging (gift from Thomas Gilmore, Boston University) were cultured in DMEM media with 10% FBS supplemented by 50 unit/ml Penicillin and 50 μg /ml Streptomycin.

### Protein samples

*In vitro* transcription/translation (IVT) samples of PU.1 (full-length, untagged) were generated using 1-Step Human Coupled IVT Kit – DNA (Thermo Fisher Scientific Cat# 88881) following the provider's instructions. Protein expression was confirmed by western analysis.

### Antibodies

PU.1 (Santa Cruz sc-352x, used for ChIP and nextPBM); C/EBPα (Santa Cruz sc-61x, used for ChIP); IRF8 (Santa Cruz sc-6058x, used for ChIP and nextPBM); human histone 3 lysine 4 mono methylation (H3K4me1) (Abcam ab8895, used for ChIP); histone 3 lysine 27 acetylation (H3K27ac) (Abcam ab177178, used for ChIP); alexa488-conjugated anti-goat (Life Technologies A11055, used for nextPBM); alexa647-conjugated anti-rabbit (Life Technologies A32733, used for nextPBM); and FLI1 (ABclonal A5644, used for nextPBM) was a gift from ABclonal.

### Plasmids

Lentiviral plasmid constructs were prepared following Feng Zhang Lab (MIT) protocol (http://genome-engineering.org/gecko/wp-content/uploads/2013/12/lentiCRISPRv2-and-lentiGuide-oligo-cloning-protocol.pdf). Briefly, to target IRF8 gene a pair of gRNAs were synthesized for exon 5 of the IRF8 gene (Primers: 5′-CACCGCTTCTGTGGACGATTACATG-3′ and 5′-AAACCATGTAATCGTCCACAGAAGC-3′) with overhangs and ligated into BsmBI digested pLentiCRISPRv2.0.

### Nuclear extracts

5 × 10^6^ THP-1 cells were pelleted at 500 × g for 5 min at 4°C in a 15 ml conical tube. The pellet was resuspended and washed twice with PBS. Cell pellet was resuspended in 1 ml of ‘low-salt buffer’ (10 mM HEPES (pH 7.9), 1.5 mM MgCl_2_, 10 mM KCl plus 1 μl protease inhibitor cocktail (Sigma-Aldrich, cat # P8340) and incubated for 10 min on ice. 50 μl of 5% IGEPAL (Sigma-Aldrich, cat # I8896) was added to the cell suspension and vortexed for 10 seconds. Released nuclei were pelleted at 750 × g for 5 min at 4°C. The supernatant was saved as the ‘cytosolic fraction’. To wash the remaining cytosolic proteins from the surface of the nuclear pellet, 100 μl of the low-salt buffer was gently pipetted onto the side of the tube and allowed to wash the pellet, making sure to not disrupt the pellet. This wash was then gently transferred to the cytosolic fraction without dislodging the nuclear pellet. 200 μl of ‘high-salt buffer’ (20 mM HEPES (pH 7.9), 25% glycerol, 1.5 mM MgCl_2_, 0.2 mM EDTA, 420 mM NaCl plus 1 μl protease inhibitor cocktail) was pipetted on the pellet and the tube went through a vigorous vortex for 30 s followed by nutation at 4°C for 1 h. The nuclei were pelleted at 4°C for 20 min at 21 000 × g. The supernatant was transferred into another tube as the nuclear soluble protein fraction. Final nuclear extract samples used in nextPBM assays were 9.6 mg/ml.

### CRISPR-mediated IRF8-knockout in THP-1 cells

To generate Lenti-CRISPR viruses, HEK293T cells were seeded in a 10 cm dish at 75% confluence a day before transfection. The next day, the confluent cells were co-transfected with 4μg of pCMV-VSV-G, 2 μg pCMV-ΔR8.91 and 1 μg plentiCRISPR v2- gRNA using a Lipofectamin-3000 kit and following the provider's instructions. The transfection mixture replaced by fresh media after 6 h and the virus-containing supernatant was collected after 48 h. Virus was concentrated by ultracentrifugation at 50 000 × g for 3 h at 4°C. The viral pellet was re-suspended in 500 μl complete medium (RPMI, 10% FBS) with 8 μg/ml Polybren and added to one million THP-1 cells in a microcentrifuge tube with 1.5 ml of complete media and shaken at 150 rpm for 30 min at room-temperature, followed by centrifugation at 850 × g for 30 min at 32°C. The THP-1 cell pellet was re-suspended in 2 ml of complete medium and was seeded in a 3 cm dish and incubated at 37°C with 5% CO_2_ for 6 days. At day 6, infected cells were selected in 0.5 μg/ml puromycin (final concentration). The media was exchanged with fresh complete media containing 0.5 μg/ml puromycin every four to six days and for total of 30 days. Cell confluence was maintained between 3 × 10^5^ cells/ml to 9 × 10^5^ cells/ml through the selection procedure and the culture volume was scaled up as necessary. Knockout efficiency in the pool of the infected cells was defined by western analysis.

### Nuclear extract treatments

*Immune depletion of IRF8 –* 7.5 μg of IRF8 antibody (abcam, ab207418) was added to 300 μL of diluted THP-1 nuclear extract (2 mg/ml total protein in nextPBM binding buffer (described below), 115 mM NaCl). The mixture was nutated at 4°C for 1 h. 75 μl of Dynabeads^®^ Protein A slurry (Thermo Fisher Scientific, 10001D) was washed once using 1 ml of nextPBM binding buffer with 115 mM salt and collected by DynaMag magnet (ThermoFisher Scientific, cat # 12321D). Collected beads were re-suspended in the nuclear extract plus antibody mixture and transferred onto HulaMixer (ThermoFisher Scientific cat # 15920) to be rotated at 4°C for 2 h at 25 rpm. DynaMag magnet was used to collect the beads and the remaining nuclear extract was checked for the depletion of IRF8 by Western analysis. *Phosphatase treatment –* A general phosphatase (Lambda protein phosphatase kit, New England Biolabs, p0753) was added to 300 μl of diluted THP-1 nuclear extract (2 mg/ml total protein in nextPBM binding buffer (described below), 115 mM NaCl), and the reaction was carried out according to the provider's instructions. Phosphatase efficiency was checked by western analysis for phospho-RNA polymerase II (abcam 5131).

### Chromatin immunoprecipitation (ChIP-seq)

Soluble chromatin was prepared from 4×10^7^ THP-1 cells according to previously described protocols (13) with some modifications (outlined below). Briefly, cells were crosslinked with 1% formaldehyde (final concentration) (Fisher Scientific, cat # F79-500) for 10 min at room temperature with gentle shaking. Crosslinking was stopped by adding 125 mM final concentration of glycine solution in PBS. Fixed cells were pelleted at 800 × g for 5 min at 4°C and washed twice with 10 ml of cold PBS in a 15 ml conical tube and pelleted at 800 × g for 5 min at 4°C. Washed cell pellet was re-suspended in 10 ml of Lysis Buffer 1 (13), nutated for 10 min at 4°C, and pelleted at 2000 × g for 5 min at 4°C. The same procedure was repeated with lysis buffer 2 (13) at room temperature followed by pelleting at 2000 × g for 5 min at 4°C. To release nuclei from hard-to-disrupt THP-1 membranes, cells were re-suspended in 10 ml of Lysis Buffer 3 (13) and were shaken vigorously (225 rpm) at room temperature for 30 min. Cells were then passed through an 18-gauge needle (VWR, cat # BD305195) 25 times using a 10ml syringe. Nuclei were pelleted at 3000 × g for 20 min at 4°C and re-suspended in 500 μl of Lysis Buffer 3 and then transferred into a 1.5 ml microfuge tube placed in Benchtop 1.5 ml Tube Cooler (Active Motif, cat # 53076). The nuclei were sonicated using Active Motif Q120AM sonicator with a 3.2 mm Probe (Active motif cat # 53053) at 25% amplitude for 15 min with 20 s ON and 30 s OFF cycles (45 cycles total). Cell debris was pelleted at 21 000 × g for 30 min at 4°C. 50 μl of the combined soluble chromatin was saved to be used as the input DNA upon reverse-crosslinking. For immunoprecipitation, 500 μl of the soluble chromatin was mixed with 30 μg of either PU.1, C/EBPα, H3K4me1 or H3K27ac antibodies (60 μg of IRF8 antibody was mixed with 1 ml of the soluble chromatin), and tubes were rotated at 25 rpm for one hour at 4°C using HulaMixer (ThermoFisher Scientific cat # 15920). 125 μl of the protein A Dynabead slurry (ThermoFisher Scientific cat # 10001D) per each rabbit antibody (PU.1. C/EBPα, H3K4me1 or H3K27ac), and 250 μl of the protein G Dynabead slurry (ThermoFisher Scientific cat # 10003D) for the goat-IRF8 antibody, were transferred into 1.5 ml microfuges and placed on DynaMag magnet (ThermoFisher Scientific, cat # 12321D) until all beads collected on the side of tubes. The solution was gently aspirated off from each tube and the beads were re-suspended in 1 ml of the Lysis Buffer 3 with several gentle inversions; beads were re-pelleted using the magnet and the lysis buffer was aspirated. Beads were then re-suspended in 50 μl of Lysis Buffer 3 and returned to HulaMixer to rotate at 35 rpm overnight at 4°C. Beads were collected and washed 6 times with 1 ml of the Lysis Buffer 3 and two times with 1 ml of the Wash Buffer (RIPA). All ChIP samples along with the 50 μl of the soluble chromatin were reverse-crosslinked by adding 200 μl of the Elution buffer and 3 μl of 20 mg/ml Proteinase K (ThermoFisher Scientific, cat # AM2546) and incubated at 65°C for overnight. Beads were collected and the solutions were transferred into a new 1.5 microfuge tube containing 1 μl of 10 mg/ml RNase A (ThermoFisher Scientific, cat # EN0531) and left at room temperature for an hour. The ChIP and input DNA were purified using QIAquick PCR Purification Kit (QIAGEN, cat # 28104) and eluted in 50 μl of 50°C Nuclease-Free Water (Thermo Fisher Scientific, AM9932). The concentration and size distribution of the ChIP-DNA samples were defined using Agilent 2100 Bioanalyser. DNA libraries were prepared using NEBNext Ultr II DNA Library Prep kit (NEB, E7645S) following the provider's instruction manual. Amplified libraries were Bioanalyzed again to check the size selection efficiency and to define the concentrations of libraries before preparing the library pool involving the same molarity of each library and sequenced by Illumina HiSeq 4000. An additional biological replicate for IRF8 (and corresponding input DNA) was sequenced using the Illumina NextSeq 500.

### ChIP-seq analysis

ChIP-seq reads were aligned to the human reference genome (hg19) using Bowtie2 (14). Aligned reads were filtered for high quality and uniquely mappable reads (MAPQ > 30) using samtools (15). Peak calling for TFs was performed using MACS2 (16) with relaxed parameters on single experiments (*P*-value < 0.01) and peaks were filtered using the irreproducible discovery rate (IDR < 0.05) across biological duplicates (17). Peak calling for histone marks was performed using MACS2 (16) with relaxed parameters on single experiments (*P*-value < 0.01) and experiments were filtered requiring identification in both biological duplicates (i.e. IDR was not used for histone marks analysis). Peaks were further filtered if they occurred in the ENCODE consortium blacklisted regions. Peak intersections were computed using bedtools (18) by first merging the peaks from all transcription factor ChIP-seq experiments into continuous genomic loci and identifying which TF(s) contained a peak within this union set. Raw and processed ChIP-seq data is available in the NCBI GEO database (Accession: GSE123872).

### Motif discovery and scoring

*De novo* motifs within peak sets were discovered using HOMER (19) (parameters: -size given -noweight -nlen 0 -len 6,8,10,12,14,16 -S 5) and subsequently used for motif scoring across all peaks. We also performed *de novo* motif analysis using MEME (20) (meme-chip parameters: -dna -meme-mod zoops) and found consistent motifs (Supplementary Figure S1). Log-odds scoring thresholds determined by HOMER against a set of random background sequences were used as significance thresholds for motif scanning. Motif scans on individual peaks were performed using a custom R script that implements the same scoring scheme as HOMER and reports the maximum log-odds score in each peak (available on Github: https://github.com/david-bray/nextPBM-paper). Uniform background probability for each nucleotide (0.25) at each position was used for log-odds scoring. We chose a uniform base-frequency background model to be consistent with that used by the HOMER algorithm, and to better support our biophysical interpretation of the nextPBM data, that is based solely on the contribution of each base to binding affinity. Motif logos were generated using the ggseqlogo R package (21). Motifs and thresholds used for ChIP-seq analysis and PBM microarray design are provided (Supplementary File 2).

### PBM design

PBM experiments were performed using custom-designed microarrays (Agilent Technologies Inc. AMADID 085624 and 085106, 8 × 60K format). 2615 PU.1 binding sites identified in ChIP-seq peaks were extracted from the genome as 20-bp genomic fragments and placed into a fixed position in the PBM probe sequence. For each unique probe sequence, 5 replicate probes were included in each orientation (10 probes per unique site). For select genomic seed sequences, 60 matching SNV probes were included to assay all single-nucleotide variants at the 20 positions of the binding site (Figure 1). All SNV sites were also included with 5 replicates and in each orientation (10 probes per unique SNV site). Probes for assaying binding site ablations and synthetic EICE sites were similarly included with 10 probes per unique DNA site. *Selection of binding sites from ChIP-seq data –* Binding sites were only included from PU.1 ChIP-seq peaks demonstrating high reproducibility across biological duplicates (IDR < 0.01), with the exception of probes included specifically to assay binding to ‘single-replicate’ regions. PU.1 ChIP-seq sites were categorized based on their log-odds motif score, proximity to co-factors, and enhancer state. PU.1 binding sites were selected from the PU.1 ChIP-seq peaks containing exactly one significant PU.1 site (see Motif analysis above). For the genomic loci in the ‘weak PU.1 motif’ category we identified no significant PU.1 site and, therefore, used the PU.1 site with maximum log-odds score (Figure 4). EICE sites were selected from PU.1-IRF8 co-occupied regions containing exactly one EICE site (see Motif analysis above). Co-occupancy PU.1 with co-factors (C/EBPα and/or IRF8) was determined if a highly reproducible ChIP-seq peak (IDR < 0.01) for each factor over-lapped by at least one base. A PU.1 ChIP-seq peak peak was annotated as ‘PU.1-alone’ if it was located greater than 200 bases away from the nearest cofactor ChIP-seq peak (in all experiments, including duplicates, with peaks called using relaxed parameters as detailed above). Enhancer states were annotated using histone modification ChIP-seq data from biological duplicates and publically available mRNA-seq data for THP-1 monocytes (GEO accession GSM927668). PU.1 sites were annotated as active if they occurred within 200 bases of the nearest H3K4me1 and H3K27ac peaks, and if the nearest gene was located between 2–500kb away and expressed above the median RPKM value. PU.1 sites were annotated as primed if they occurred within 200 bases of the nearest H3K4me1 peak only, and if the nearest gene was located between 2–500kb away and expressed below the median RPKM value. A full list of DNA probes used, their corresponding probe category and additional annotation can be found in the supplemental data (Supplementary File 1).

### NextPBM and PBM experiments and analysis

Microarray DNA double stranding and basic PBM protocols are as previously described (22,23). All wash steps were carried out in coplin jars on an orbital shaker at 125 rpm. Double-stranded DNA microarrays were first pre-washed in PBS containing 0.01% Triton X-100 (5 min), rinsed in a PBS bath, and then blocked with 2% milk in PBS for 1 hour. Following the blocking step, arrays were washed in PBS containing 0.1% Tween-20 (5 min), then in PBS containing 0.01% Triton X-100 (2 min), and finally briefly rinsed in a PBS bath. *Protein binding* – Arrays were then incubated with the protein sample (IVT protein or THP-1 nuclear lysate, details in Supplementary File 3) for one hour in a binding reaction buffer containing: 2% milk (final concentration); 20 mM Hepes buffer, pH 7.9; 100 mM NaCl; 1 mM DTT; 0.2 mg/mL BSA; 0.02% Triton X-100; and 0.4 mg/mL salmon testes DNA (Sigma D7656). *Primary antibody* – After protein incubation, microarrays were washed with PBS containing 0.5% Tween-20 (3 min), then in PBS containing 0.01% Triton X-100 (2 min), followed by a brief PBS rinse. Microarrays were then incubated with 10 μg/mL of primary antibody (see Supplementary File 3) in 2% milk in PBS (20 min). *Secondary antibody* - After primary antibody incubation, microarrays were washed with PBS containing 0.5% Tween-20 (3 min), then in PBS containing 0.01% Triton X-100 (2 min), followed by a brief PBS rinse. Microarrays were then incubated with 7.5 μg/mL of alexa488-conjugated secondary antibody or alexa647-conjugated secondary antibody (see Supplementary File 3) in 2% milk in PBS (20 min). Excess antibody was removed by washing with PBS containing 0.05% Tween-20 (3 min), then PBS (2 min). *PBM data analysis* - Microarrays were scanned with a GenePix 4400A scanner and fluorescence was quantified using GenePix Pro 7.2. Exported data were normalized using MicroArray LINEar Regression (7). Microarray probe sequences are provided (Supplementary File 1). PBM data analysis and SNV approach for logo generation is as previously described (24). Similarity between the DNA binding models generated using nextPBM and those from previously published studies was computed using the PWMSimilarity function from the TFBSTools R bioconductor package (25) (Supplementary Figure S2). A threshold binding *z*-score of 2.0 (at the seed probe) was imposed to ensure accurate binding models. Processed PBM z-score data is available in the supplementary data (Supplementary File 1), and all raw PBM data has been deposited in the NCBI GEO database (Accession: GSE123946). Scatterplots and boxplots were generated using the ggplot2 R package (26). Motif logos were generated using the ggseqlogo R package (21). The significance of PU.1 binding affinity and motif scores between groups was calculated using the two-sided Wilcoxon–Mann-Whitney test implemented in R.

### PU.1-IRF8 cooperativity score

PU.1-IRF8 cooperativity was scored by quantifying the deviation of the observed EICE z-scores from an extract experiment from the expected *z*-scores based on the IVT sample experiment. To define the *expected* EICE *z*-scores a second degree polynomial model was fit to the *z*-scores for the canonical PU.1 probes as follows:

\begin{equation*}\ {y_1} = {\beta _0} + {\beta _1}{x_1} + {\beta _2}x_1^2 + {\varepsilon _1}\end{equation*}

where ${y_1}$ is the vector of PU.1 z-scores observed in the extract sample, ${x_1}$ is the vector of PU.1 z-scores observed for the IVT sample, ${\beta _0}$, ${\beta _1}$ and ${\beta _2}$ are coefficients of the best-fit polynomial model and ${\varepsilon _1}$ is the vector of error terms needed to equate ${y_1}$ to the function of ${x_1}$. A polynomial model was used to fit the canonical PU.1 site z-scores in place of a linear model to allow for non-linearity due to PU.1 concentration differences between experiments.

The coefficients fit above are then used to compute the expected EICE *z*-scores for the extract experiment based on the IVT experiment z-scores:

\begin{equation*}\ {y_2} = {\beta _0} + {\beta _1}{x_2} + {\beta _2}x_2^2 + {\varepsilon _2}\end{equation*}

where ${y_2}$ is the vector of PU.1 *z*-scores observed at EICE probes in the extract sample, ${x_2}$ is the vector of PU.1 *z*-scores observed for the IVT sample, ${\varepsilon _2}$ is the vector of error terms needed to equate ${y_2}$ to the function of ${x_2}$ comprised of the coefficients fit using the canonical PU.1 probes.

The error vectors ${\varepsilon _1}$ and ${\varepsilon _2}$ are then used to compute the PU.1-IRF8 cooperativity scores:

\begin{equation*}scores\ = \frac{{|{\varepsilon _2}|}}{{variance\left( {{\varepsilon _1}} \right)}}\end{equation*}

## RESULTS

### Nuclear extract protein-binding microarrays (nextPBMs)

To demonstrate the nextPBM approach, we examined binding of PU.1/SPI1 from human monocytes as a test case. PU.1 is a master regulator of the myeloid lineage (27–29) and functions to establish localized histone modifications that define the cell-specific enhancer repertoire (19,30,31). In myeloid cells, PU.1 can bind DNA autonomously to 5′-GGAA-3′ ETS motifs, or cooperatively with IRF8 to 5′-GGAANNGAAA(C/G)-3′ ETS-IRF composite elements (EICEs) (32–34). To define PU.1 binding sites for our assay, we performed genome-wide chromatin immunoprecipitation (ChIP-seq) for PU.1 in human THP-1 monocytes, and selected 2,499 DNA sites in ChIP-positive regions that matched a PU.1 position-weight matrix (PWM) (Materials and Methods, Supplementary File 1). To identify composite PU.1-IRF8 EICE elements, we performed IRF8 ChIP-seq and selected 116 EICE sites from regions bound by both PU.1 and IRF8. Nuclear extracts from human THP-1 monocyte cells were made using a detergent-based cell lysis and extraction procedure and incubated with the double-strand DNA microarrays (Materials and Methods). As proteins in the assay are not epitope-tagged, primary antibodies were used to label PU.1, followed by fluorescently labeled secondary antibodies (Materials and Methods).

PU.1 binding was detected to genome-derived sites significantly above background sites (Figure 1B), demonstrating that there is sufficient endogenous protein in nuclear extracts to quantify TF binding using our assay. PU.1 binding profiles for individual replicate experiments were highly correlated, demonstrating high reproducibility between nextPBM experiments (Figure 1C). To assess the sensitivity of our nextPBM assay, we generated a PU.1 DNA-binding logo using a single-nucleotide variant (SNV) approach (Materials and Methods) (24). Briefly, we measured PU.1 binding to a 20 bp-long *seed* sequence and all 60 SNV sequences (Figure 1D); logos were generated from binding scores to each SNV sequence (Figure 1D). The PU.1 binding logo agreed well with the established ETS-type motif (35), demonstrating that we can accurately measure the TF binding specificity using nextPBMs. As the nuclear extract is highly heterogenous and contains other ETS family proteins, we asked whether the binding of another ETS factor could be assayed in parallel using the same DNA sites. Probing the nextPBM with antibodies to FLI1, another ETS factor expressed in THP-1 monocytes, we were able to define the FLI1 binding motif (35) using the same seed and SNV probes as used for PU.1. We note that PU.1 and FLI1 are related ETS factors and exhibit only minor differences in their DNA binding specificity, namely the 2–3 bases upstream of the 5′-GGAA-3′ core element (Figure 1D); however, we were able to resolve their distinct motifs in parallel using the SNV approach. These results show that robust and sensitive quantification of TF binding can be performed for TFs at endogenous levels in heterogeneous nuclear extracts.

### Characterizing the DNA binding of PU.1 and IRF8 in monocytes

**Figure 2.**
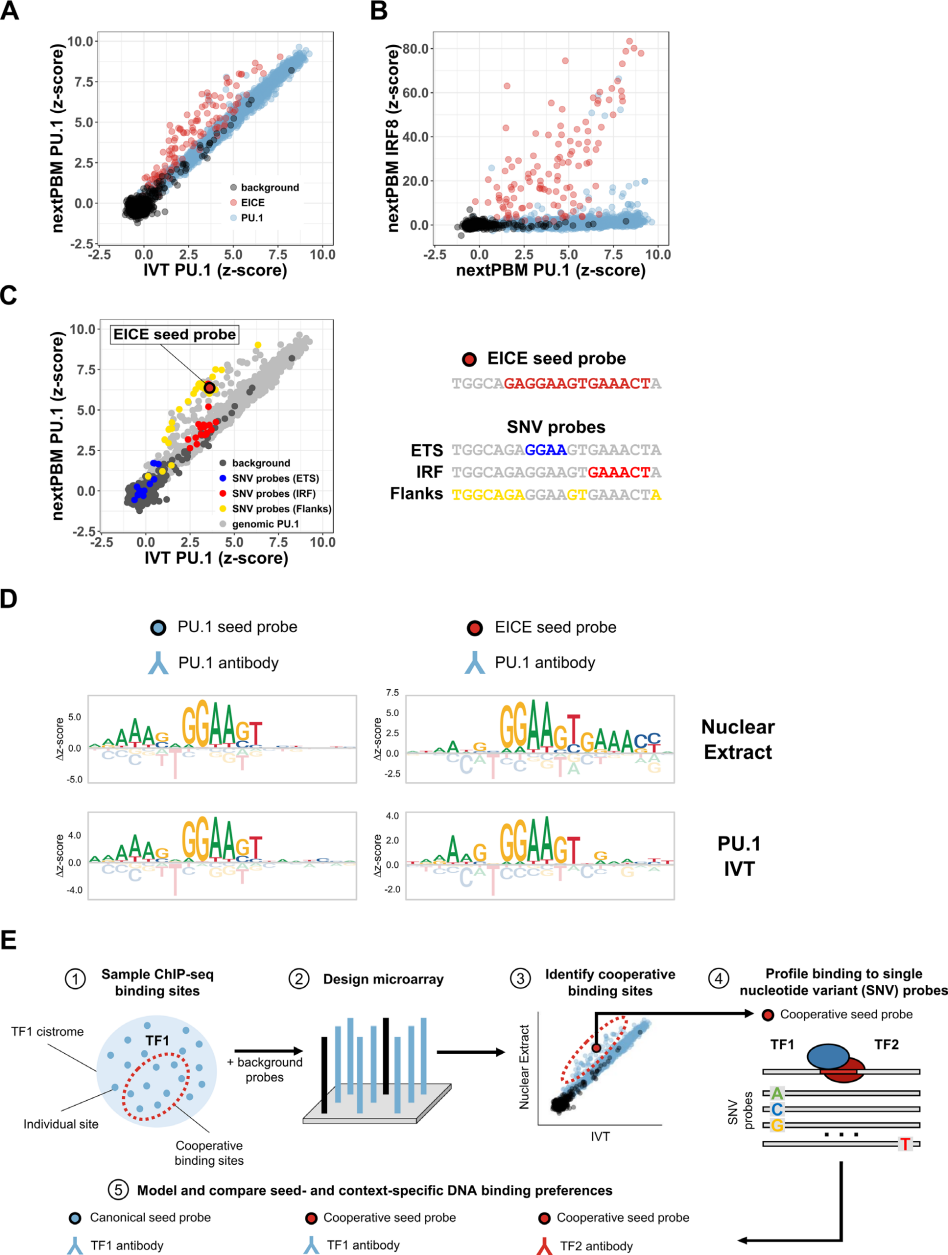
DNA sequence determinants of PU.1-IRF8 cooperative binding. (**A**) Scatterplot of PU.1 binding *z*-scores obtained from nuclear extract (nextPBM) versus in vitro transcribed/translated (IVT) PU.1 for random background probes (*n* = 500), ETS-IRF composite element (EICE) probes (*n* = 116), and canonical PU.1 probes (*n* = 2499). (**B**) Scatterplot of IRF8 binding *z*-scores in nuclear extract versus PU.1 binding z-scores in nuclear extract for the same sets of probes as in (A). (**C**) Left – scatterplot of PU.1 binding z-scores in nuclear extract versus IVT PU.1 for probes included in (A) and SNV probes corresponding to the EICE seed probe shown right. Highlighted probes correspond to SNV probes containing variations in either the ETS core half-site (blue), IRF core site (red), or flanking and linker bases (yellow). Right - schematic of EICE seed probe and bases comprising individual sub-elements. (**D**) Sequence logos obtained using a canonical PU.1 seed probe (left column) and a cooperative ETS-IRF composite element (EICE) probe (right column) from nuclear extract (top row) and from IVT PU.1 (bottom row). (**E**) Workflow schematic for identifying cooperative binding sites using nextPBM. 1 – ChIP-seq sites for a given transcription factor of interest (TF1) can be sampled and used to construct probes for a microarray design. The sample will contain sites where TF1 is cooperatively bound with other factors. 2 – TF1 sample probes are combined with a set of random background probes against which binding *z*-scores are computed to form the basis of a microarray design. 3 – Profiling binding of TF1 in nuclear extract versus IVT allows for the discovery of cooperative binding sites bound higher in nuclear extract (shown above the diagonal). 4 – Cooperative sites identified can be used as seed probes in a subsequent experiment where SNV probes are included in the microarray and profiled. 5 – Binding to SNV probes is used to model and compare seed- and context-specific DNA binding preferences of TF1 to identify composite elements and likely binding partners.

To identify monocyte-specific features of PU.1 binding, we compared monocyte nextPBM data for PU.1 with binding data using *in vitro* translated (IVT) PU.1 (Figure 2A).

Binding to genomic PU.1 sites was highly correlated between extract PU.1 and IVT PU.1 (Figure 2A, highlighted in blue); however, binding of extract PU.1 was enhanced to the EICEs present in genomic regions co-occupied by PU.1 and IRF8 (Figure 2A, highlighted in red). We confirmed that IRF8 was also bound to the EICE sites using an IRF8 nextPBM (Figure 2B). IRF8 binds almost exclusively to the EICEs, consistent with the known requirement for cooperative binding with PU.1 in monocytes. The enhanced PU.1 binding to EICEs (Figure 2A) suggests cooperative binding with a monocyte-specific cofactor. These results demonstrate that using nextPBMs to compare the TF binding profiles from nuclear extracts and purified/IVT protein provides a HT approach to identify cell-specific cooperative binding.

### Defining DNA-sequence determinants of PU.1-IRF8 cooperativity

To examine determinants of PU.1-IRF8 cooperativity we visualized the impact of SNVs on PU.1 binding (Figure 2C). We highlighted SNVs that occur in different regions of an EICE site: ETS/PU.1 half-site (blue); IRF half-site (red); flanking and linker sequence (yellow) (Figure 2C). SNVs in the ETS half-site abrogate PU.1 binding for both IVT and nuclear extract samples as expected (Figure 2C, blue). SNVs in the IRF half-site affect the cooperative binding but do not affect the binding of IVT PU.1, capturing the impact of IRF8 present in the extract samples (Figure 2C, red). SNVs in the flanking and linker sequence affect PU.1-IRF8 complex affinity but largely do not abrogate the cooperative interactions (i.e., most yellow data points are above the diagonal), demonstrating that cooperative binding does not require specific sequence features outside of the core half-sites (Figure 2C, yellow). This analysis highlights that nextPBMs can be used to dissect the determinants of cooperativity for a single DNA binding site.

Binding specificity can also be visualized as DNA-binding logos, providing a way to easily reveal binding differences to distinct classes of DNA sites under different sample conditions (Figure 2D). PU.1 binding logos generated for a seed sequence that was not bound cooperatively match canonical PU.1 logos for both the nuclear extract and IVT experiments (Figure 2D, left). In contrast, the PU.1 binding logos for a cooperatively bound seed sequence differ between the conditions: the logo from the nuclear extract experiment resembles the composite EICE element, showing the influence of the IRF8 binding, while the logo from the IVT experiment shows just the PU.1 logo (Figure 2D, right). We note that we obtain consistent motifs when using other high-scoring seed sequences (Supplementary Figures S3 and S4). The impact of cofactors on binding to the distinct classes of DNA sites can be easily visualized using SNV-based logo analysis. Using this approach we can analyze multiple TF binding modes in parallel in a single experiment (i.e., the PU.1 logos for cooperative and non-cooperative binding were determined using a single experiment).

### Approach to identify and characterize cooperative binding

Our results provide an approach for the identification and characterization of cell-specific cooperative binding (Figure 2E). Briefly, putative DNA binding sites of a TF can be identified from genomic data (e.g. ChIP-seq, or ATAC-seq combined with motif analysis, etc.) or be designed synthetically based on prior knowledge, and can be incorporated into a nextPBM microarray (Figure 2E, steps 1 and 2). For example, scanning PU.1 ChIP-seq data with a PU.1 position-weight matrix (PWM) with relaxed cutoff scores can be used to identify both autonomous and cooperatively bound sites. Next, comparison of binding profiles between nuclear extract and purified TF experiments can be used to identify cooperatively bound sites (Figure 2E, step 3). Based on this data, one can design SNV probes for target DNA sites and perform a follow-up nextPBM experiment to define DNA-binding logos that reveal the cooperative binding specificity and provide information about the identity of cooperatively acting factors. For example, monitoring PU.1 binding revealed the 5′-GAAACT-3′ IRF logo (Figure 2D), which could be matched to PWMs from databases to make predictions about the PU.1 cooperative binding partner. The outlined approach provides a HT assay to identify and characterize cooperative TF complexes in a cell-specific manner.

### Sensitivity of cooperative binding to nuclear extract concentration

**Figure 3.**
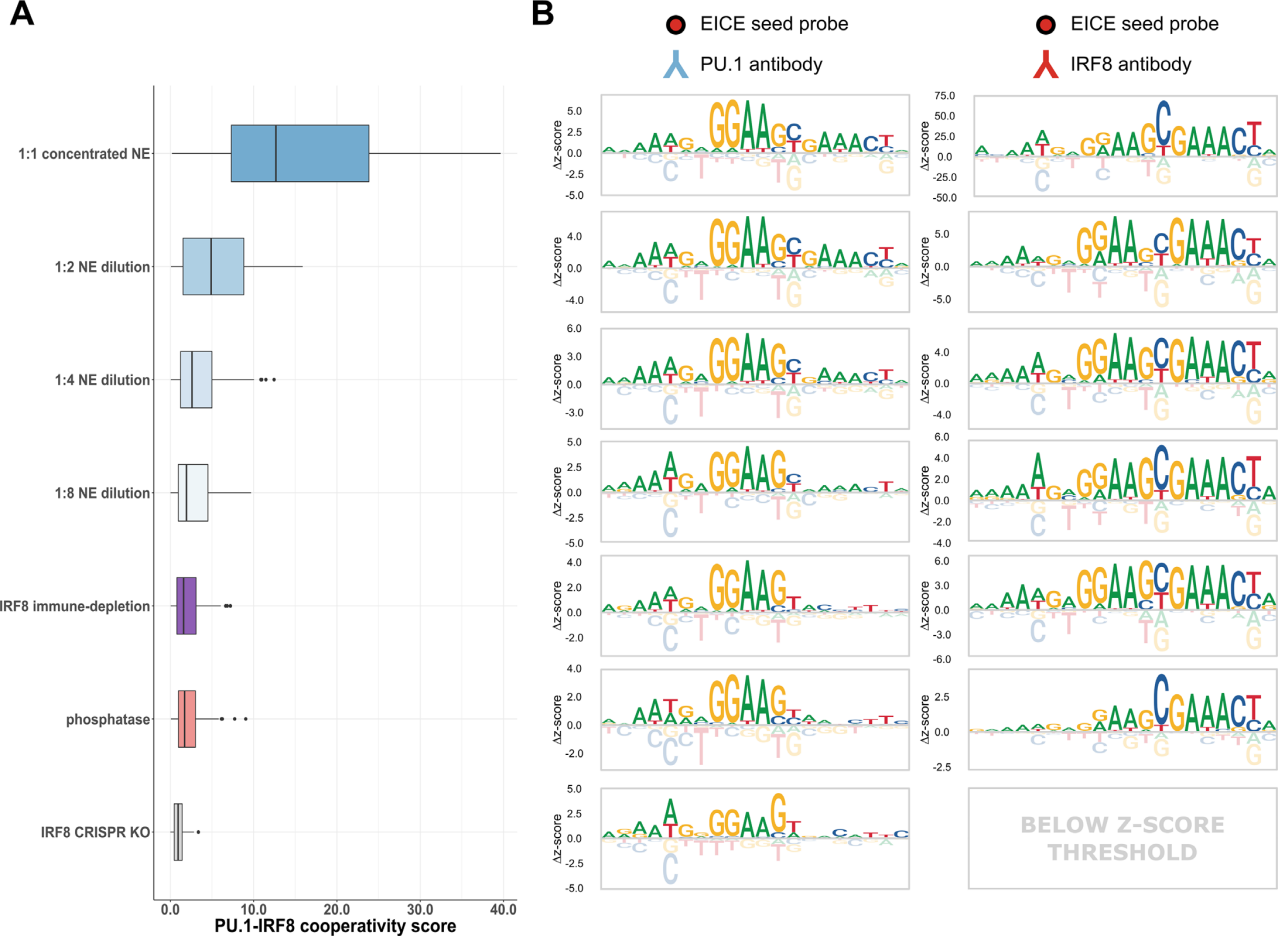
Effects of different nuclear extract treatments on PU.1-IRF8 cooperative binding. (**A**) Boxplot of PU.1-IRF8 cooperativity scores (see Methods) for a set of EICE probes (*n* = 116) in various listed nuclear extract (NE) conditions and treatments including a gradient of 2-fold dilutions (1:1, 1:2, 1:4, and 1:8), an extract where IRF8 has been immune-depleted (IRF8 immune-depletion), an extract treated with a broad-spectrum phosphatase (phosphatase), and an extract generated from a line of cells where IRF8 has been knocked out (IRF8 CRISPR KO). Boxplot elements – center line: median, box limits: first and third quartiles, whiskers: 1.5x interquartile range, individual points: data points beyond end of whiskers. (**B**) Sequence logos obtained by profiling PU.1 binding (left column) and IRF8 binding (right column) to the same sample EICE seed probe in the corresponding nuclear extract treatments/conditions from (A).

To test the sensitivity of our results on nuclear extract concentration, we performed nextPBM experiments at successive dilutions of monocyte nuclear extract. We quantified PU.1 cooperativity as the off-diagonal displacement of the 116 EICE sites from the autonomously bound PU.1 sites (as in Figure 2A, Materials and Methods). We found that PU.1-IRF8 cooperativity decreased with decreasing extracts concentrations (Figure 3A). We also assessed cooperativity by monitoring the PU.1 DNA binding logo for an EICE site as extract concentration varied. We observed a consistent PU.1 element (i.e. 5′-GGAA-3′ core) with a successively weaker IRF8 element (i.e., 5′-GAACT-3′) (Figure 3B, left). As PU.1 can bind to DNA in an autonomous or cooperative fashion, both the bound PU.1 and PU.1-IRF8 complexes contribute to the microarray spot intensity in a PU.1 nextPBM. Therefore, observing PU.1 cooperativity requires that the increase in spot intensity due to the presence of PU.1-IRF8 complexes must be discerned beyond the signal intensity from PU.1 binding alone, leading to the observed concentration dependence in our assay. In contrast, IRF8 is an obligate dimer; therefore, all signal in an IRF8 nextPBM is due to PU.1-IRF8 complexes. As such, the binding logos for an IRF8 nextPBM are much more robust to extract concentrations and we can discern cooperative EICE logos for all extract concentrations (Figure 3B, right). The results demonstrate that the concentration dependence of cooperative binding in our assay will depend on the characteristics of the individual binding partner.

### Assessing the impact of cofactors and PTMs

To determine whether the cooperative PU.1 binding to EICEs was solely due to IRF8 we used CRISPR/Cas9 to mutate the IRF8 gene in THP-1 monocyte cells and performed nextPBM using nuclear extracts from IRF8-deficient cells. Cooperative binding of PU.1 was lost in the absence of IRF8 protein (Figure 3A and B, bottom), consistent with reduced PU.1 ChIP-seq to EICEs reported for Irf8-null mouse macrophages (36). CRISPR/Cas9-based deletion of target TFs remains a labor-intensive process; therefore, we sought to develop a more rapid approach for testing the impact of cofactors on cooperative TF binding. We developed an immune-depletion (ID) protocol to deplete a TF from the nuclear extracts in the nextPBM pipeline (Figure 1A). NextPBM with IRF8-ID extracts showed similar abrogation of the enhanced PU.1 binding (Figure 3A and B), corroborating the CRISPR/Cas9-based results that IRF8 is solely responsible for PU.1 cooperativity. Our ID step removed >90% of the IRF8 from the extract sample (Supplementary Figure S5); however, an IRF8 nextPBM was still successful and we were able to generate an EICE logo, demonstrating that for obligate heterodimers such as IRF8, cooperative binding can be detected even with low levels of protein in the extract. NextPBM with an ID treatment provides rapid assay for the impact of cofactor proteins on cooperative TF complexes.

PTMs play a central role in the regulation of TF function and cooperative TFs complexes *in vivo*. Cooperative binding of PU.1 and IRF8 has been reported to involve phosphorylation of IRF8 (37). To test the impact of phosphorylation on PU.1 cooperativity we incubated our extract sample with a broad-spectrum phosphatase prior to the nextPBM (Figure 1A, Supplementary Figure S6, Materials and Methods). Phosphatase treatment of our extract samples abrogated PU.1 cooperative binding to the EICEs (Figure 3A), showing the dependence of PU.1-IRF8 cooperativity on phosphorylation. The disruption of cooperative binding can also be seen in the PU.1 binding logo as an absence of the IRF8 half-site (Figure 3B). We note that this treatment had no effect on autonomous PU.1 binding (data not shown). Therefore, nextPBM with an enzymatic treatment of the extract provides a rapid assay for the PTM-dependence of TF binding to diverse DNA sequences.

### Screening synthetic cooperative elements

**Figure 4.**
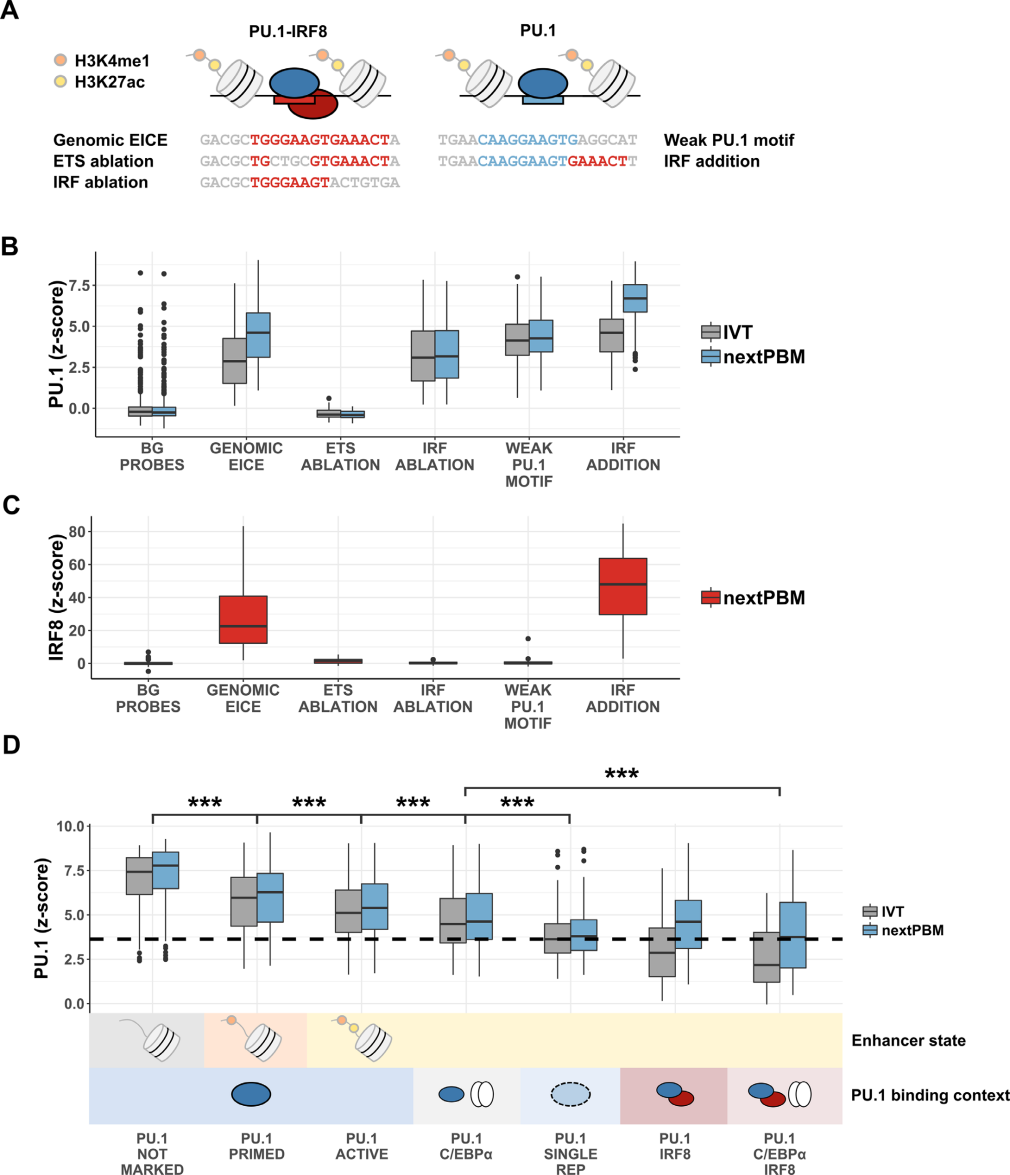
Screening synthetic cooperative elements and binding from different genomic contexts. (**A**) Schematic showing representative probe sequences and corresponding mutated elements. For each genomic EICE from active enhancers in our array design (*n* = 60), there is a corresponding probe with the ETS and IRF core sites independently mutated to contain a different *k*-mer. For each canonical PU.1 probe with a weak motif (n = 199), there is a corresponding probe with an IRF half-site added. (**B**) Distributions of PU.1 binding z-scores for DNA probe groups in (A) in nuclear extract (blue) compared to IVT. BG – random background probe set (*n* = 500). Boxplot elements – center line: median, box limits: first and third quartiles, whiskers: 1.5× interquartile range, individual points: data points beyond end of whiskers. (**C**) Distributions of IRF8 binding *z*-scores to the same DNA probe groups as in A and B. Boxplot elements: same as in (B). (**D**) Distributions of PU.1 binding z-scores for DNA probe categories defined by ChIP-seq co-occupancy with cofactors (PU.1 binding context) and/or histone modifications (enhancer state). ‘NOT MARKED’ indicates the absence of H3K4me1 and H3K27ac histone modifications. ‘SINGLE REP’ designates a category of probes designed using PU.1 ChIP-seq peaks that were discovered in a single biological replicate but were not observed in a duplicate experiment. The dashed black line denotes an approximate ChIP-seq reproducibility threshold corresponding to the median z-score obtained for the PU.1 SINGLE REP group in IVT. Boxplot elements: same as in (A) and (B).

NextPBMs present an opportunity to screen synthetic DNA elements (i.e., mutant or novel sequences) for cooperative TF binding in a more cell-native context that may be used to probe the rules of cooperativity or to design synthetic genetic regulatory elements. We first tested our ability to screen for the impact of half-site ablations on cooperative binding. We compared the binding of PU.1 and IRF8 to 60 EICE elements and matched mutants with an ablated ETS or IRF site (Figure 4A and B). Mutating the ETS half-site abrogates PU.1 binding, whereas mutating the IRF half-site only affects the observed cooperativity (Figure 4B). In contrast, IRF8 binding is abrogated with mutations to either the ETS or the IRF half-site (Figure 4C). These results demonstrate that IRF8 binding is dependent on cooperativity with PU.1, but not *vice versa*, consistent with observations *in vivo* (32–34). We next tested our ability to screen for new cooperative sites and generated 199 synthetic EICEs by combining low-affinity PU.1 sites with a consensus IRF8 site (Figure 4A). An adjacent IRF8 site greatly enhanced PU.1 binding to all sites in the presence of the nuclear extract but not for IVT PU.1 (Figure 4B). Similarly, IRF8 bound strongly to these synthetic EICEs, and at levels higher than seen for the genomic EICEs (Figure 4C). NextPBMs provide a platform for HT screening of DNA sequences for cooperative binding that can account for the impact of the native cell-specific protein environment.

### Binding affinity of PU.1 correlates with enhancer state and cofactor occupancy

To examine how nextPBM data can inform genomic analysis of TF binding, we examined PU.1 binding to sites from genomic regions defined by distinct chromatin states and cofactor occupancy. In addition to IRF8, PU.1 functions with C/EBPα to bind chromatin and establish macrophage-specific genes expression (19,38–40). PU.1 does not bind DNA cooperatively with C/EBPα; rather they function collaboratively through mutual effects on repressive chromatin environments. We performed ChIP-seq on C/EBPα and identified PU.1 binding sites in regions co-occupied by both PU.1 and C/EBPα, or by all three factors (PU.1, C/EBPα and IRF8). To examine the relation between chromatin state on PU.1 binding, we also performed ChIP-seq for histone 3 lysine 4 mono-methylation (H3K4me1) and H3K27 acetylation (H3K27ac) that define poised (H3K4me1 only) and active (H3K4me and H3K27ac) enhancer states (41,42).

We first examined PU.1 binding to distinct enhancer states: primed, active, or unmarked (no H3K4me1 or H3K27ac marks) (Figure 4D). To control for the effect of cofactors we limited our analysis to sites from PU.1-only occupied regions. PU.1 binding affinity shows a clear trend with enhancer state. High-affinity PU.1 binding to unmarked loci is in agreement with previous studies (43) and suggests that PU.1 occupancy to less biophysically accessible chromatin regions requires high-affinity sites. Low-affinity PU.1 binding in active enhancers reveals that functional PU.1 sites are not the highest affinity, and that genome-wide analyses of the highest affinity TF sites may be enriched for non-functional binding. Binding to all sites agrees between the nuclear extract and IVT samples, suggesting that there is no influence of cooperative binding to these genomic elements and that the binding trends are defined by autonomous PU.1 binding.

We next examined PU.1 binding at active enhancers co-occupied by collaborative (C/EBPα) or cooperative (IRF8) cofactors (Figure 4D). We observe a clear trend in affinity for the PU.1 IVT data that suggests an impact of cofactors on PU.1 binding. First, PU.1 binding sites are lower affinity in regions co-occupied by either cofactor than in regions occupied by PU.1 alone (Figure 4D). For example, in regions co-occupied with C/EBPα, PU.1 binding sites have Δ*z*-scores ∼ 0.5 lower than for PU.1-only regions (*P*-value < 0.001), and in regions co-occupied with IRF8 the affinity is even lower (Δ*z*-score ∼ 2.0, *P*-value < 0.001). Unexpectedly, in regions co-occupied by both cofactors (C/EBPα and IRF8) PU.1 binding is the lowest affinity (Δ*z*-score ∼ 2.5, *P*-value < 0.001), suggesting that the effects of collaborative and cooperative cofactors on PU.1 binding are independent and additive. However, when analyzing the nextPBM data, we observe that cooperativity with IRF8 significantly increases the PU.1 binding to EICE sites. For perspective, we examined PU.1 binding to sites from genomic regions identified as PU.1-bound in only a single ChIP-seq replicate experiment (‘single-replicate regions’), which we found to be lower affinity than for reproducible PU.1 ChIP sites. We observe that, in the absence of IRF8, PU.1 affinity falls below this ‘reproducible level’, which may explain the drop in PU.1 ChIP-seq signal observed in IRF8 knock-out mouse macrophages (36). Our results demonstrate that cooperative binding with IRF8 or collaborative function with C/EBPα allow PU.1 binding sites to be much lower affinity than an optimal site, and highlight the perspective gained by analyzing TF binding using both purified/IVT and nuclear extract samples.

## DISCUSSION

HT throughput methods for characterizing TF-DNA binding provide critical biophysical data for genomic analyses of gene regulation (1–3). Cell-specific PTMs (11,12) and cofactors (1,44) can affect TF binding, but are not implicitly accounted for in current HT methods. Here we describe the nextPBM methodology for the characterization of protein-DNA binding that uses nuclear extracts to account for the impact of cell-specific PTMs and cofactors. We show that a direct comparison of binding profiles between nuclear extract of purified/IVT samples can reveal cooperative binding activity and cooperatively bound sites. Using an SNV-based approach to query sequence specificity and generate binding logos we can examine binding and cooperativity for individual genomic sites. The flexibility to analyze binding specificity for individual sites allows multiple binding modes to be directly studied in parallel in a single experiment. This approach is analogous to the seed-and-wobble approach previously described for universal PBMs that quantify TF binding to k-mers (7). We note that DNA shape is known to play an important role in TF binding specificity (45–47), and future studies that examines the role of DNA shape in the context of multi-protein complexes and cell-specific extracts will be informative. We anticipate that this approach will be particularly useful when studying TFs that function as obligate heterodimers and may have multiple binding partners in a complex nuclear extract and, therefore, interact with DNA using distinct binding modes. To address the impact of cofactors and phosphorylation on TF binding we have incorporated immune-depletion and phosphatase-treatment steps into our nextPBM pipeline. Incorporation of additional enzymatic treatment steps will allow us to expand our assay to study other PTMs (e.g., demethylases to study impact of methylation, etc.). NextPBMs provide an extendible platform to study the DNA binding of endogenous TF complexes in a cell-specific manner. We anticipate that nextPBM-based comparison of cell-specific TF binding and cooperative assembly will be particularly informative when applied to comparisons of different cell types, cell-stimulation conditions, and to cells from disease contexts.

Our study outlines a new approach to identify cooperative binding TFs and cooperatively bound sites. First, by sampling from bound genomic loci identified by ChIP-seq experiments, one can design a nextPBM microarray to survey a diverse set of binding sites for a TF. Currently, using available microarray platforms (Materials and Methods), and accounting for replicate probes, we can assay up to ∼18 000 unique genomic sites in an experiment, which is sufficient to thoroughly sample (or even completely cover) most TF cistromes. Direct comparison of nextPBM binding profiles from nuclear extract and purified protein can then reveal differentially bound DNA sites. Enhanced binding in nextPBM experiments indicates potential cooperative binding, and by reanalyzing these binding sites with a subsequent SNV-based array design we can generate binding logos on a per-site basis that can be used to make predictions about the possible cooperative binding partners. The identity of binding partners can then be tested using nextPBMs with an immune-depletion step. Using nextPBMs to compare the binding profiles of TFs from different cells will be particularly useful for studying the TFs that function as obligate heterodimers and may utilize different partner proteins in different cell types. While the cooperative complex examined in this manuscript involves two proteins (PU.1 and IRF8), this approach can, in principle, be used to examine cooperative assembly of more than two proteins as all constituents are available in the nuclear extract. We have previously demonstrated that cooperative complexes of more than two purified proteins can be assayed using the PBM technology (4). This approach to identify cooperative binding can also be used to screen novel DNA elements for cooperative binding activity (Figure 4), providing a HT method for the design and testing of cell-specific cooperative elements that can be used to construct synthetic gene regulatory elements for mammalian cells.

We examined the binding of PU.1 and IRF8 from human monocytes, and identified the known composite EICE binding logos using nextPBMs probing either PU.1 or IRF8. Using CRISPR/Cas9-based IRF8 knockout and immune-depletion we demonstrated that IRF8 is the only cooperative binding partner for PU.1 in human monocytes. Investigating the relationship between binding, cooperativity and genomic occupancy we found that PU.1 binding affinity exhibits a clear trend with both enhancer type and cofactor co-occupancy. We found that the highest affinity PU.1 sites are in genomic regions not containing the H3K4me and H3K27ac histone modifications for active enhancers, and lowest affinity sites are in active enhancers. Furthermore, co-occupancy with either collaborating (C/EBPα) or cooperative (IRF8) cofactors correlated with lower affinity binding sites, suggesting that cofactor occupancy allows for the evolutionary selection of lower affinity binding sites. Surprisingly, coincident binding of PU.1 with both C/EBPα and IRF8 allowed for still lower affinity sites to be utilized. These results highlight that functional binding sites are not the highest affinity, and that genomic analyses biased to high affinity may miss functionally relevant sites. Finally, comparing binding profiles for nextPBM and IVT samples across stratified genomic sites demonstrated that PU.1 binding was autonomous on all sites except for the EICEs where it was cooperative with IRF8. NextPBM-based binding analysis of genome-derived sites provides insights into the biophysical determinants of TF binding. We anticipate that similar studies that compare TF binding profiles from different cellular conditions will provide new insights into the mechanisms of cell-specific binding and gene regulation.

## DATA AVAILABILITY

ChIP-seq and nextPBM/PBM data from this study were deposited in GEO (Accession: GSE123947). Custom motif scanning scripts written for this study are available on Github (https://github.com/david-bray/nextPBM-paper).

## Supplementary Material

Supplementary Data

## References

[1] Siggers T., Gordân R. Protein-DNA binding: complexities and multi-protein codes. Nucleic Acids Res. 2013; 42:2099–2111. 24243859 10.1093/nar/gkt1112 PMC3936734

[2] Slattery M., Zhou T., Yang L., Dantas Machado A.C., Gordân R., Rohs R. Absence of a simple code: how transcription factors read the genome. Trends Biochem. Sci. 2014; 39:381–399. 25129887 10.1016/j.tibs.2014.07.002 PMC4149858

[3] Andrilenas K.K., Penvose A., Siggers T. Using protein-binding microarrays to study transcription factor specificity: homologs, isoforms and complexes. Brief Funct. Genomics. 2015; 14:17–29. 25431149 10.1093/bfgp/elu046 PMC4366590

[4] Siggers T., Duyzend M.H., Reddy J., Khan S., Bulyk M.L. Non-DNA-binding cofactors enhance DNA-binding specificity of a transcriptional regulatory complex. Mol. Syst. Biol. 2011; 7:555. 22146299 10.1038/msb.2011.89 PMC3737730

[5] Slattery M., Riley T., Liu P., Abe N., Gomez-Alcala P., Dror I., Zhou T., Rohs R., Honig B., Bussemaker H.J. et al. Cofactor binding evokes latent differences in DNA binding specificity between Hox proteins. Cell. 2011; 147:1270–1282. 22153072 10.1016/j.cell.2011.10.053 PMC3319069

[6] Jolma A., Yin Y., Nitta K.R., Dave K., Popov A., Taipale M., Enge M., Kivioja T., Morgunova E., Taipale J. DNA-dependent formation of transcription factor pairs alters their binding specificity. Nature. 2015; 527:384–388. 26550823 10.1038/nature15518

[7] Berger M.F., Philippakis A.A., Qureshi A.M., He F.S., Estep P.W., Bulyk M.L. Compact, universal DNA microarrays to comprehensively determine transcription-factor binding site specificities. Nat. Biotechnol. 2006; 24:1429–1435. 16998473 10.1038/nbt1246 PMC4419707

[8] Badis G., Berger M.F., Philippakis A.A., Talukder S., Gehrke A.R., Jaeger S.A., Chan E.T., Metzler G., Vedenko A., Chen X. et al. Diversity and complexity in DNA recognition by transcription factors. Science. 2009; 324:1720–1723. 19443739 10.1126/science.1162327 PMC2905877

[9] Jolma A., Yan J., Whitington T., Toivonen J., Nitta K.R., Rastas P., Morgunova E., Enge M., Taipale M., Wei G. et al. DNA-binding specificities of human transcription factors. Cell. 2013; 152:327–339. 23332764 10.1016/j.cell.2012.12.009

[10] Fang B., Mane-Padros D., Bolotin E., Jiang T., Sladek F.M. Identification of a binding motif specific to HNF4 by comparative analysis of multiple nuclear receptors. Nucleic Acids Res. 2012; 40:5343–5356. 22383578 10.1093/nar/gks190 PMC3384313

[11] Tootle T.L., Rebay I. Post-translational modifications influence transcription factor activity: a view from the ETS superfamily. Bioessays. 2005; 27:285–298. 15714552 10.1002/bies.20198

[12] Filtz T.M., Vogel W.K., Leid M. Regulation of transcription factor activity by interconnected post-translational modifications. Trends Pharmacol. Sci. 2014; 35:76–85. 24388790 10.1016/j.tips.2013.11.005 PMC3954851

[13] Lee T.I., Johnstone S.E., Young R.A. Chromatin immunoprecipitation and microarray-based analysis of protein location. Nat. Protoc. 2006; 1:729–748. 17406303 10.1038/nprot.2006.98 PMC3004291

[14] Langmead B., Salzberg S.L. Fast gapped-read alignment with Bowtie 2. Nat. Methods. 2012; 9:357–359. 22388286 10.1038/nmeth.1923 PMC3322381

[15] Li H., Handsaker B., Wysoker A., Fennell T., Ruan J., Homer N., Marth G., Abecasis G., Durbin R. 1000 Genome Project Data Processing Subgroup The Sequence Alignment/Map format and SAMtools. Bioinformatics. 2009; 25:2078–2079. 19505943 10.1093/bioinformatics/btp352 PMC2723002

[16] Zhang Y., Liu T., Meyer C.A., Eeckhoute J., Johnson D.S., Bernstein B.E., Nusbaum C., Myers R.M., Brown M., Li W. et al. Model-based analysis of ChIP-Seq (MACS). Genome Biol. 2008; 9:R137. 18798982 10.1186/gb-2008-9-9-r137 PMC2592715

[17] Landt S.G., Marinov G.K., Kundaje A., Kheradpour P., Pauli F., Batzoglou S., Bernstein B.E., Bickel P., Brown J.B., Cayting P. et al. ChIP-seq guidelines and practices of the ENCODE and modENCODE consortia. Genome Res. 22:1813–1831. 10.1101/gr.136184.111 PMC3431496 22955991

[18] Quinlan A.R., Hall I.M. BEDTools: a flexible suite of utilities for comparing genomic features. Bioinformatics. 2010; 26:841–842. 20110278 10.1093/bioinformatics/btq033 PMC2832824

[19] Heinz S., Benner C., Spann N., Bertolino E., Lin Y.C., Laslo P., Cheng J.X., Murre C., Singh H., Glass C.K. Simple combinations of lineage-determining transcription factors prime cis-regulatory elements required for macrophage and B cell identities. Mol. Cell. 2010; 38:576–589. 20513432 10.1016/j.molcel.2010.05.004 PMC2898526

[20] Bailey T.L., Boden M., Buske F.A., Frith M., Grant C.E., Clementi L., Ren J., Li W.W., Noble W.S. MEME SUITE: tools for motif discovery and searching. Nucleic Acids Res. 2009; 37:W202–W208. 19458158 10.1093/nar/gkp335 PMC2703892

[21] Wagih O. ggseqlogo: a versatile R package for drawing sequence logos. Bioinformatics. 2017; 33:3645–3647. 29036507 10.1093/bioinformatics/btx469

[22] Berger M.F., Bulyk M.L. Universal protein-binding microarrays for the comprehensive characterization of the DNA-binding specificities of transcription factors. Nat. Protoc. 2009; 4:393–411. 19265799 10.1038/nprot.2008.195 PMC2908410

[23] Siggers T., Gilmore T.D., Barron B., Penvose A. Characterizing the DNA binding site specificity of NF-κB with protein-binding microarrays (PBMs). Methods Mol. Biol. 2015; 1280:609–630. 25736775 10.1007/978-1-4939-2422-6_36

[24] Andrilenas K.K., Ramlall V., Kurland J., Leung B., Harbaugh A.G., Siggers T. DNA-binding landscape of IRF3, IRF5 and IRF7 dimers: implications for dimer-specific gene regulation. Nucleic Acids Res. 2018; 46:2509–2520. 29361124 10.1093/nar/gky002 PMC5861432

[25] Tan G., Lenhard B. TFBSTools: an R/bioconductor package for transcription factor binding site analysis. Bioinformatics. 2016; 32:1555–1556. 26794315 10.1093/bioinformatics/btw024 PMC4866524

[26] Wickham H. ggplot2. 2016; Springer.

[27] Nerlov C., Graf T. PU.1 induces myeloid lineage commitment in multipotent hematopoietic progenitors. Genes Dev. 1998; 12:2403–2412. 9694804 10.1101/gad.12.15.2403 PMC317050

[28] Rosenbauer F., Tenen D.G. Transcription factors in myeloid development: balancing differentiation with transformation. Nature Reviews Immunology. 2007; 7:105–117. 10.1038/nri2024 17259967

[29] Scott E.W., Simon M.C., Anastasi J., Singh H. Requirement of transcription factor PU.1 in the development of multiple hematopoietic lineages. Science. 1994; 265:1573–1577. 8079170 10.1126/science.8079170

[30] Ghisletti S., Barozzi I., Mietton F., Polletti S., De Santa F., Venturini E., Gregory L., Lonie L., Chew A., Wei C.-L. et al. Identification and characterization of enhancers controlling the inflammatory gene expression program in macrophages. Immunity. 2010; 32:317–328. 20206554 10.1016/j.immuni.2010.02.008

[31] Barozzi I., Simonatto M., Bonifacio S., Yang L., Rohs R., Ghisletti S., Natoli G. Coregulation of transcription factor binding and nucleosome occupancy through DNA features of Mammalian Enhancers. Mol. Cell. 2014; 54:844–857. 24813947 10.1016/j.molcel.2014.04.006 PMC4048654

[32] Rehli M., Poltorak A., Schwarzfischer L., Krause S.W., Andreesen R., Beutler B. PU.1 and interferon consensus sequence-binding protein regulate the myeloid expression of the human Toll-like receptor 4 gene. J. Biol. Chem. 2000; 275:9773–9781. 10734131 10.1074/jbc.275.13.9773

[33] Eklund E.A., Jalava A., Kakar R. PU.1, interferon regulatory factor 1, and interferon consensus sequence-binding protein cooperate to increase gp91(phox) expression. J. Biol. Chem. 1998; 273:13957–13965. 9593745 10.1074/jbc.273.22.13957

[34] Meraro D., Hashmueli S., Koren B., Azriel A., Oumard A., Kirchhoff S., Hauser H., Nagulapalli S., Atchison M.L., Levi B.Z. Protein-protein and DNA-protein interactions affect the activity of lymphoid-specific IFN regulatory factors. J. Immunol. 1999; 163:6468–6478. 10586038

[35] Wei G.-H., Badis G., Berger M.F., Kivioja T., Palin K., Enge M., Bonke M., Jolma A., Varjosalo M., Gehrke A.R. et al. Genome-wide analysis of ETS-family DNA-binding in vitro and in vivo. EMBO J. 2010; 29:2147–2160. 20517297 10.1038/emboj.2010.106 PMC2905244

[36] Mancino A., Termanini A., Barozzi I., Ghisletti S., Ostuni R., Prosperini E., Ozato K., Natoli G. A dual cis-regulatory code links IRF8 to constitutive and inducible gene expression in macrophages. Genes Dev. 2015; 29:394–408. 25637355 10.1101/gad.257592.114 PMC4335295

[37] Sharf R., Meraro D., Azriel A., Thornton A.M., Ozato K., Petricoin E.F., Larner A.C., Schaper F., Hauser H., Levi B.Z. Phosphorylation events modulate the ability of interferon consensus sequence binding protein to interact with interferon regulatory factors and to bind DNA. J. Biol. Chem. 1997; 272:9785–9792. 9092512 10.1074/jbc.272.15.9785

[38] Feng R., Desbordes S.C., Xie H., Tillo E.S., Pixley F., Stanley E.R., Graf T. PU.1 and C/EBPalpha/beta convert fibroblasts into macrophage-like cells. Proc. Natl. Acad. Sci. U.S.A. 2008; 105:6057–6062. 18424555 10.1073/pnas.0711961105 PMC2327209

[39] Laiosa C.V., Stadtfeld M., Xie H., de Andres-Aguayo L., Graf T. Reprogramming of committed T cell progenitors to macrophages and dendritic cells by C/EBP alpha and PU.1 transcription factors. Immunity. 2006; 25:731–744. 17088084 10.1016/j.immuni.2006.09.011

[40] Xie H., Ye M., Feng R., Graf T. Stepwise reprogramming of B cells into macrophages. Cell. 2004; 117:663–676. 15163413 10.1016/s0092-8674(04)00419-2

[41] Heintzman N.D., Stuart R.K., Hon G., Fu Y., Ching C.W., Hawkins R.D., Barrera L.O., Van Calcar S., Qu C., Ching K.A. et al. Distinct and predictive chromatin signatures of transcriptional promoters and enhancers in the human genome. Nat. Genet. 2007; 39:311–318. 17277777 10.1038/ng1966

[42] Creyghton M.P., Cheng A.W., Welstead G.G., Kooistra T., Carey B.W., Steine E.J., Hanna J., Lodato M.A., Frampton G.M., Sharp P.A. et al. Histone H3K27ac separates active from poised enhancers and predicts developmental state. Proc. Natl. Acad. Sci. U.S.A. 2010; 107:21931–21936. 21106759 10.1073/pnas.1016071107 PMC3003124

[43] Pham T.H., Minderjahn J., Schmidl C., Hoffmeister H., Schmidhofer S., Chen W., Langst G., Benner C., Rehli M. Mechanisms of in vivo binding site selection of the hematopoietic master transcription factor PU.1. Nucleic Acids Res. 2013; 41:6391–6402. 23658224 10.1093/nar/gkt355 PMC3711439

[44] Garvie C.W., Wolberger C. Recognition of specific DNA sequences. Mol. Cell. 2001; 8:937–946. 11741530 10.1016/s1097-2765(01)00392-6

[45] Andrabi M., Hutchins A.P., Miranda-Saavedra D., Kono H., Nussinov R., Mizuguchi K., Ahmad S. Predicting conformational ensembles and genome-wide transcription factor binding sites from DNA sequences. Sci. Rep. 2017; 7:4071. 28642456 10.1038/s41598-017-03199-6 PMC5481346

[46] Zhou T., Shen N., Yang L., Abe N., Horton J., Mann R.S., Bussemaker H.J., Gordân R., Rohs R. Quantitative modeling of transcription factor binding specificities using DNA shape. Proc. Natl. Acad. Sci. U.S.A. 2015; 112:4654–4659. 25775564 10.1073/pnas.1422023112 PMC4403198

[47] Yang L., Zhou T., Dror I., Mathelier A., Wasserman W.W., Gordân R., Rohs R. TFBSshape: a motif database for DNA shape features of transcription factor binding sites. Nucleic Acids Res. 2014; 42:D148–55. 24214955 10.1093/nar/gkt1087 PMC3964943

