# Supplementary material for: NextPBM: a platform to study cell-specific transcription factor binding and cooperativity
Source: Nucleic Acids Res. 2019 Jan 18;47(6):e31. doi: 10.1093/nar/gkz020 (PMC6451091; doi:10.1093/nar/gkz020)
Supplement: Supplementary Data [file gkz020_supplemental_files.zip › supplementary_information_v2.docx]

**SUPPLEMENTARY INFORMATION**


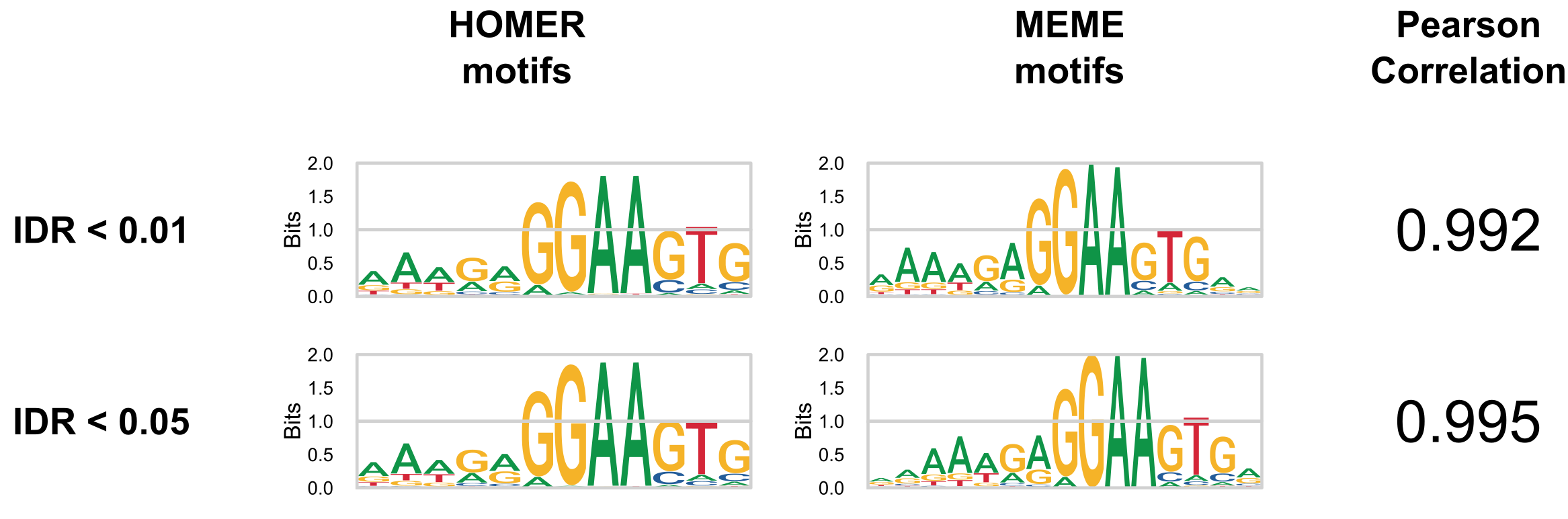


**Supplementary Figure 1. PU.1 de novo motifs obtained using HOMER and MEME**

HOMER and MEME (meme-chip) de novo motifs for PU.1 ChIP-seq peaks with cross-replicate IDR < 0.01 (top row) and IDR < 0.05 (bottom row). Pearson correlation summarizing the similarity between the HOMER and MEME motifs for a given IDR threshold is shown.


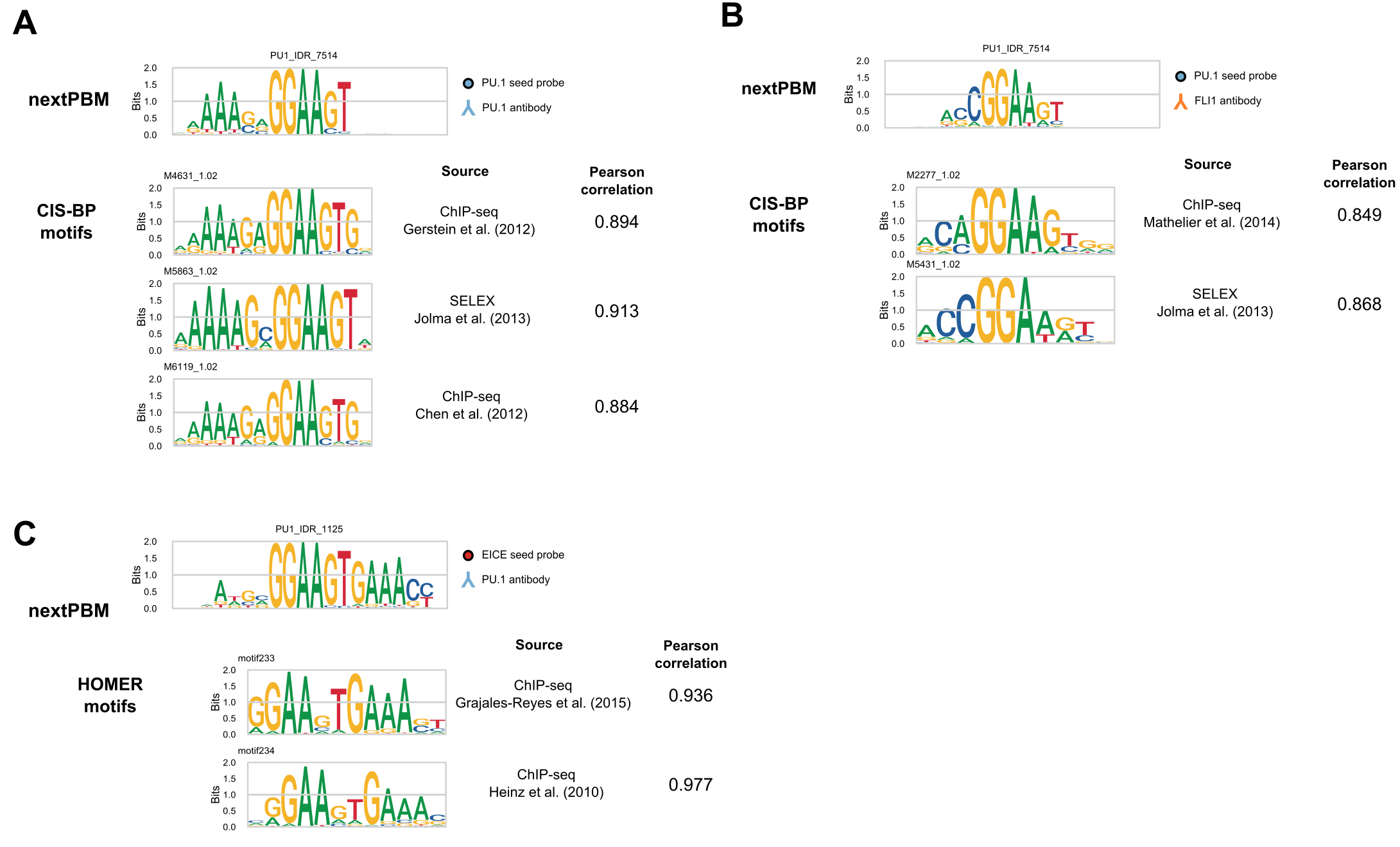


**Supplementary Figure 2. Similarity between nextPBM binding models and existing database entries**

**(A)** Similarity between a representative PU.1 binding model obtained with nextPBM using the single nucleotide variant probe approach (see **Methods**) and PU.1 binding models obtained in previously published *in vivo* (ChIP-seq) and *in vitro* (SELEX) investigations (CIS-BP motifs). Similarity is measured using the maximum Pearson correlation computed between the nextPBM position-weight matrix (PWM) and the database PWM. Label above the nextPBM binding model corresponds to the identifier of the ChIP-seq peak from which the PU.1 seed probe was selected (see **Supplementary File 1**).

**(B)** Similarity between an FLI1 binding model generated as in (A) and FLI1 binding models obtained in previous investigations. Similarity is computed as in (A).

**(C)** Similarity between a representative PU.1 binding model using an ETS-IRF composite element (EICE) seed probe and characterized EICEs from previous investigations. Similarity is computed as in (A) and (B).


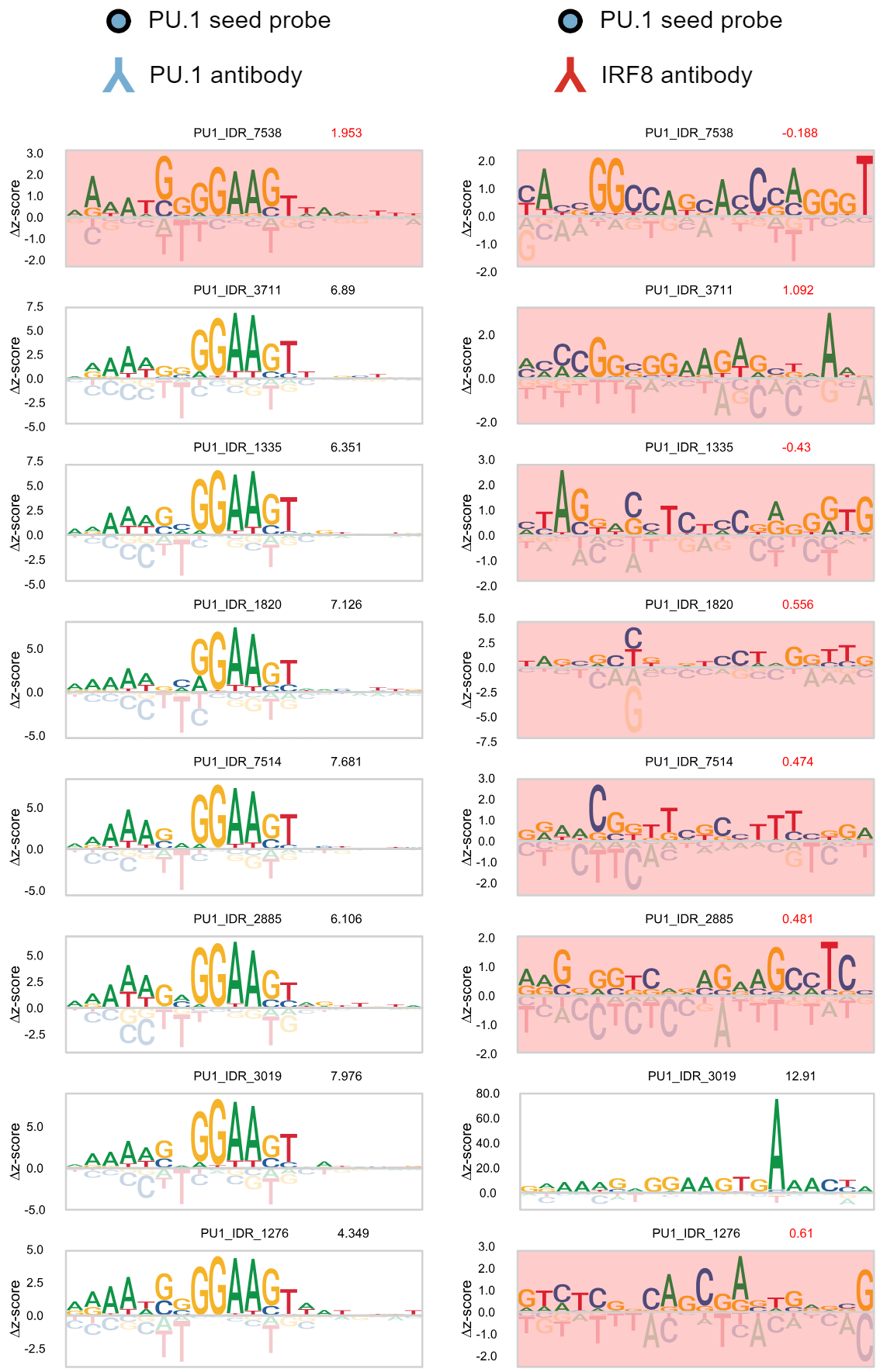


**Supplementary Figure 3. PU.1 and IRF8 binding models at canonical PU.1 seed probes**

Left: nextPBM PU.1 binding models obtained using binding of PU.1 to 8 canonical seed probes and each of the single nucleotide variants of the seed sequence. Label above the model corresponds to the identifier of the ChIP-seq peak from which the PU.1 seed probe was selected. The score shown to the top right of each model is the PU.1 binding z-score obtained at the seed probe. Low-scoring models (binding with z-score < 2.0), where PU.1 does not bind well to the seed probe are highlighted in red and the corresponding model is tinted red. The Δz-score values are computed relative to the positional median score (see **Methods**).

Right: IRF8 binding models obtained using the same 8 canonical PU.1 seeds. Low-scoring models (also with z-score < 2.0), where IRF8 does not bind well to the seed probe are also tinted red.


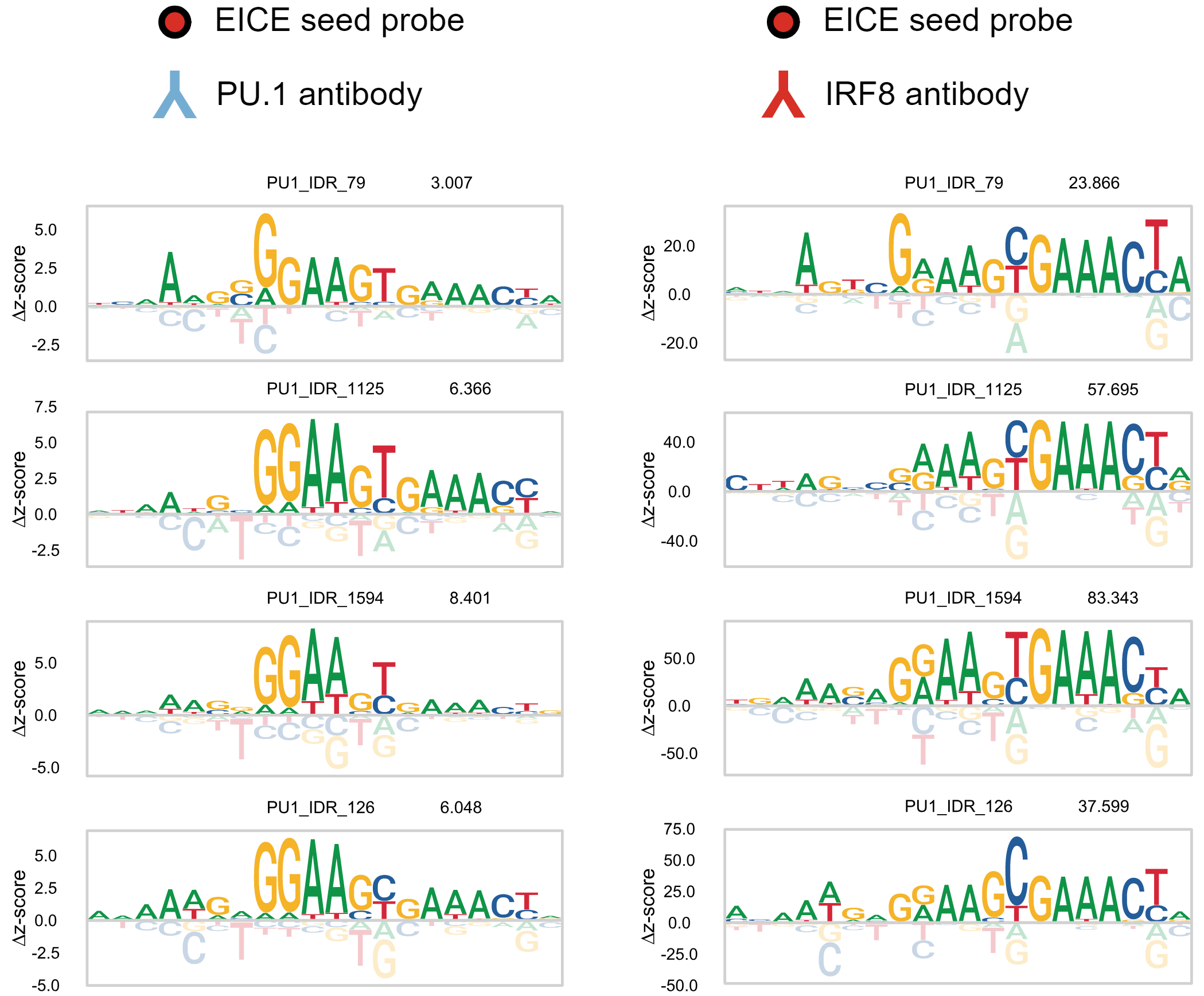


**Supplementary Figure 4. PU.1 and IRF8 binding models at ETS-IRF composite element (EICE) seed probes**

Left: PU.1 binding models obtained using binding of PU.1 to 4 EICE seed probes and each of the single nucleotide variants of the seed sequence. Labels and z-scores are shown as in Supplementary Figure 3. Identical z-score thresholds are used as in Supplementary Figure 3.

Right: IRF8 binding models obtained using the same 4 EICE seeds.


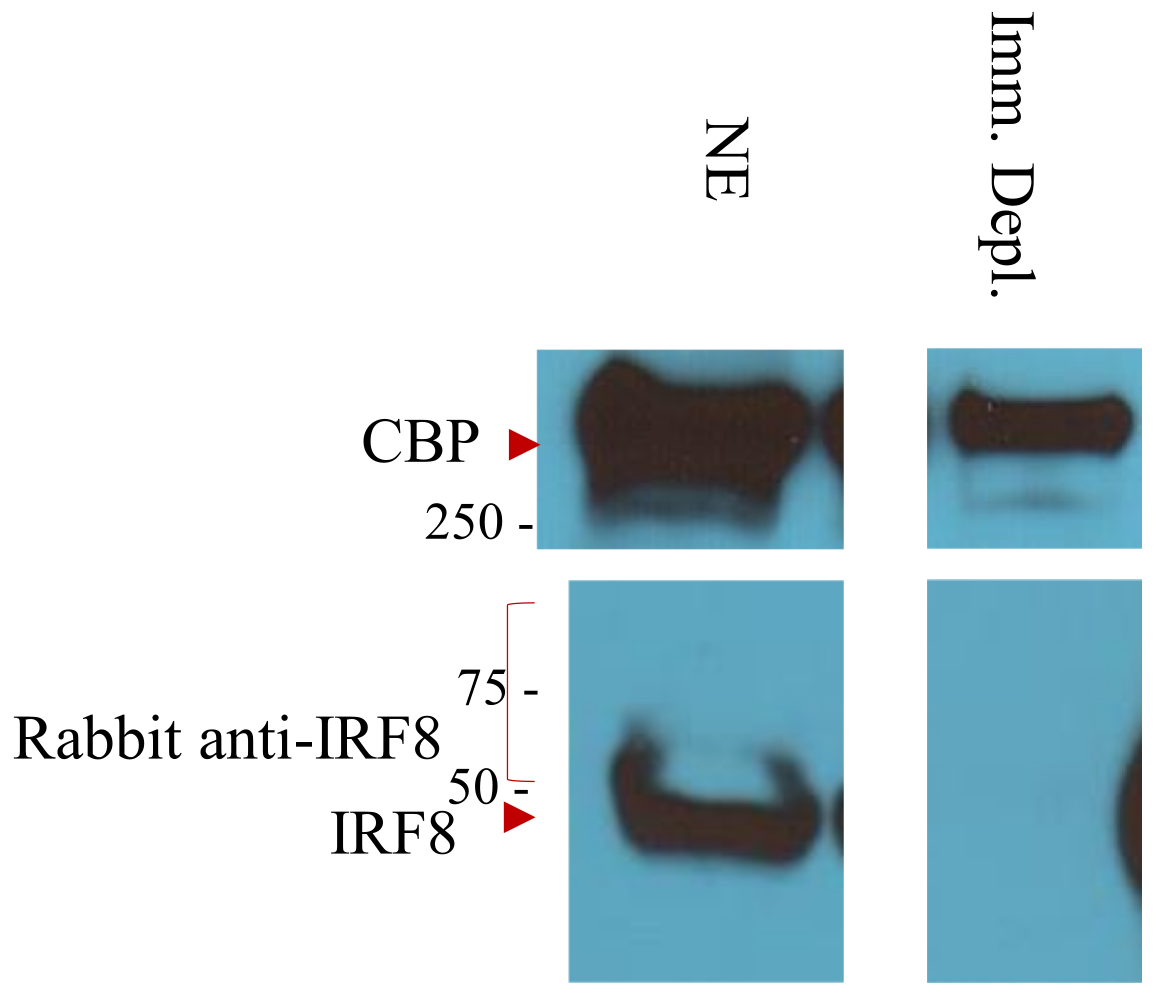


**Supplementary Figure 5. Immune-depletion of IRF8 from nuclear extract**

Western blot comparing IRF8 protein levels in untreated nuclear extract (NE) to NE where IRF8 has been immune-depleted (Imm. Depl.). CBP protein levels were used as a loading control. Each sample includes 60µg of total nuclear extract protein. The Western blot has been cropped for clarity.


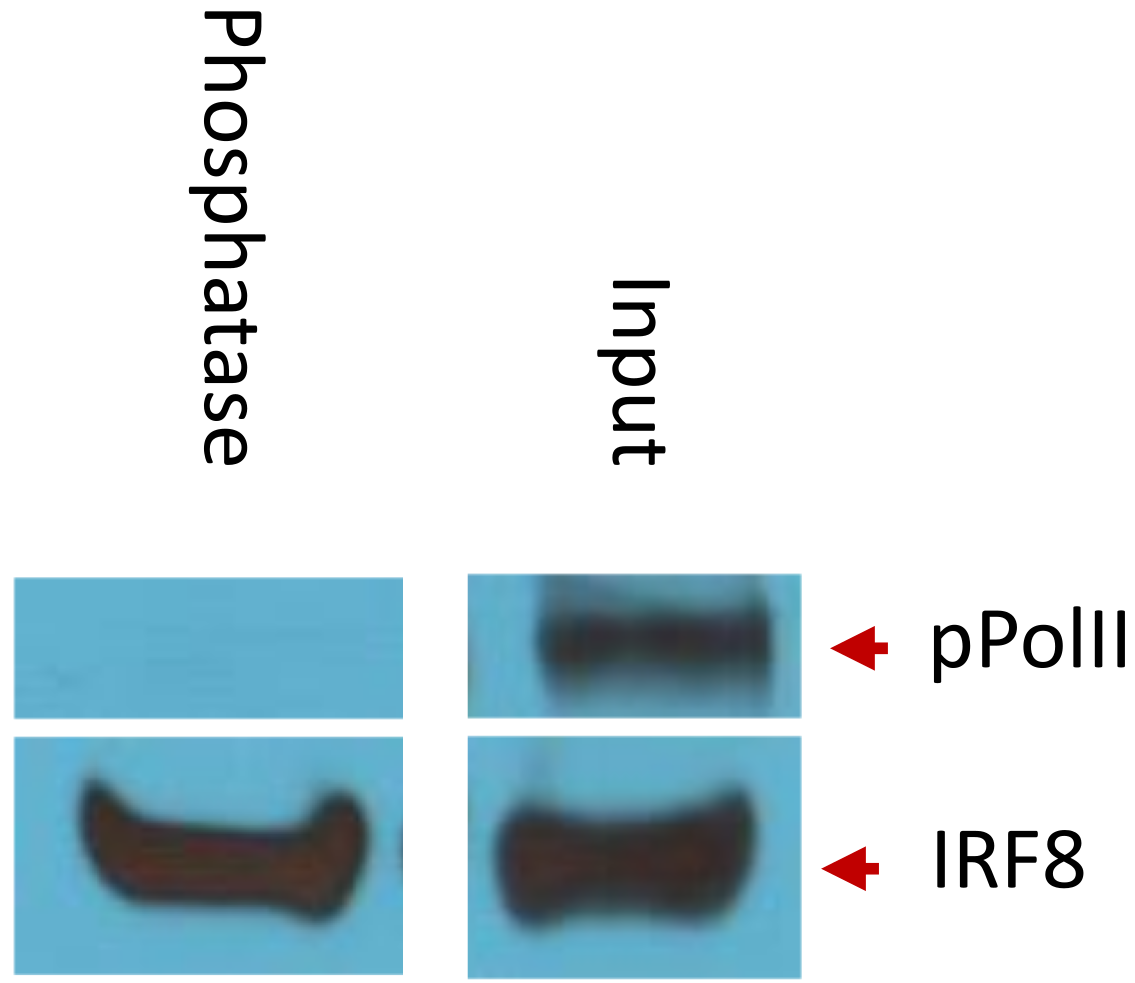


**Supplementary Figure 6. Phosphatase assay validation**

Western blot comparing protein levels of phosphorylated RNA polymerase II (pPolII) in untreated nuclear extract (NE) to nuclear extract treated with broad-spectrum phosphatase. IRF8 protein levels were used as a loading control. The Western blot has been cropped for clarity.

**Supplementary File 1. NextPBM/PBM Data**

**Supplementary File 2. Transcription Factor Motifs and Thresholds**

**Supplementary File 3. NextPBM/PBM Experimental Details**
